# Associations between C-reactive protein-triglyceride glucose index and asthma risk among Chinese adults: evidence from the CHARLS cohort and external hospital-based validation

**DOI:** 10.3389/fimmu.2026.1837567

**Published:** 2026-09-28

**Authors:** Chun-Fa Zhang, Qiao-Yuan Fei, Bei-Jing Cheng

**Affiliations:** 1 Department of Respiratory and Critical Care Medicine, Jinjiang Municipal Hospital (Shanghai Sixth People's Hospital Fujian), Quanzhou, Fujian, China; 2 Wusong Central Hospital, Shanghai, China; 3 National Centre for Epidemiology and Population Health, The Australian National University, Canberra, ACT, Australia; 4 School of Public Health, Southeast University, Nanjing, Jiangsu, China

**Keywords:** asthma, C-reactive protein–triglyceride glucose index, insulin resistance, prospective cohort, systemic inflammation

## Abstract

**Background:**

Inflammation and insulin resistance are important contributors to asthma pathogenesis. However, the association between the C-reactive protein–triglyceride glucose index (CTI), reflecting both systemic inflammation and insulin resistance, and asthma risk remains insufficiently characterized.

**Methods:**

A total of 10,234 adults aged ≥45 years were included from the China Health and Retirement Longitudinal Study (CHARLS). The association between CTI and incident asthma was assessed using multivariable Cox proportional hazards models and restricted cubic spline (RCS) analyses, with subgroup analyses performed to explore potential effect modification. An independent hospital-based case–control dataset (277 cases and 277 matched controls) was used for external validation using conditional logistic regression and RCS analyses. Additionally, among patients with asthma in the validation dataset, the associations of CTI with eosinophil and neutrophil counts were evaluated using multivariable linear regression models.

**Results:**

During a median follow-up of 84 months, 436 incident asthma cases occurred among 10,234 middle-aged and older adults (incidence, 4.26%), with a mean baseline CTI level of 4.76. After adjustment for potential confounders, higher CTI was consistently associated with an increased risk of asthma, with per 1-unit increase corresponding to a 24% higher risk (HR = 1.24, 95% CI: 1.06–1.45). RCS analyses indicated an approximately linear dose–response relationship. Subgroup analyses showed the associations were more pronounced among participants aged 45–49 years, males, unmarried individuals, and never drinkers. Furthermore, analyses in the hospital-based validation population generally supported the main findings. In the validation dataset, among adults with asthma, per 1-unit increase in CTI was associated with an 8.00% increase in eosinophil counts (95% CI: 4.58%–11.53%) and a 16.72% increase in neutrophil counts (95% CI: 8.24%–25.86%).

**Conclusions:**

Higher CTI levels were associated with increased asthma risk in adults and elevated eosinophil and neutrophil counts among adults with asthma.

## 1 Introduction

Asthma is a chronic inflammatory airway disease with hyperresponsiveness and variable airflow limitation, which may become partially irreversible over time (1, 2). Asthma affects nearly 300 million people worldwide, with prevalence varying from about 1% to 18% across different populations and regions (2, 3). In 2019, asthma claimed around 461,000 lives worldwide, representing the second-highest mortality among chronic respiratory diseases (4). Despite declines in age-standardized incidence and mortality rates over recent decades, the absolute number of asthma cases continues to increase, largely driven by population growth and aging (5, 6). In China, the burden of asthma is particularly pronounced, with an estimated prevalence of 4.2% among adults, corresponding to more than 45 million affected individuals (7). Therefore, identifying modifiable risk factors and/or early markers is essential for the primary prevention of asthma.

Airway and systemic inflammation play a central role in the pathophysiology of asthma, and multiple inflammatory biomarkers have been investigated for their association with asthma risk and severity. Commonly studied inflammatory biomarkers include interleukins (e.g., IL-6, IL-8, IL-13), fractional exhaled nitric oxide (FeNO), eosinophil counts, and acute-phase proteins (8, 9). C-reactive protein (CRP), a stable and widely measured acute-phase protein, has emerged as an important biomarker in asthma research, reflecting low-grade systemic inflammation that correlates with asthma risk and severity of exacerbations (10, 11). Metabolism also plays a critical role as a potential risk factor in respiratory diseases, affecting both susceptibility and disease progression (12). Insulin resistance (IR), defined as a reduced physiological response to insulin, is a common pathological mechanism underlying multiple metabolic disorders and has recently been implicated in respiratory diseases, including asthma (13, 14). The triglyceride–glucose (TyG) index, introduced in 2008 as a simple and reliable surrogate marker of IR (15), has been widely applied in clinical research. Recent studies suggested that elevated TyG levels were associated with an increased risk of asthma, particularly among adolescents (16, 17). Overall, inflammation and IR are important drivers of asthma pathogenesis. Composite indices integrating these markers may provide a more comprehensive assessment of asthma risk.

In this context, the C-reactive protein–triglyceride glucose index (CTI), proposed by Ruan et al. in 2022 (18), was developed to integrate systemic inflammation and IR and has attracted increasing attention in recent research. While CTI has been associated with various health outcomes, including cardiovascular disease (CVD) (19–22), its relationship with asthma remains insufficiently characterized. To date, only two recent studies have investigated the association between CTI and asthma prevalence; however, these studies were limited by their cross-sectional designs, lack of external validation using independent clinical data, and limited evaluation of asthma-related inflammatory biomarkers (23, 24). To address these gaps, we utilized longitudinal data from the China Health and Retirement Longitudinal Study (CHARLS) to investigate the association between CTI and incident asthma risk, validate these findings using an independent hospital-based dataset, and explore the associations between CTI and inflammatory biomarkers among adults with asthma.

## 2 Methods

### 2.1 Study design and population

Participants were drawn from the CHARLS, a nationally representative cohort of Chinese adults aged ≥45 years. The study was established in 2011–2012, with follow-up waves conducted in 2013, 2015, 2018, and 2020. For the present analysis, we included individuals enrolled at the 2011 baseline and followed through 2018, as these waves have been fully harmonized and quality-controlled by the CHARLS team (https://charls.charlsdata.com/pages/Data/harmonized_charls/zh-cn.html). Participants completed face-to-face computer-assisted personal interviews (CAPI), underwent standardized anthropometric measurements, and provided biological samples for laboratory analyses. Although CHARLS primarily targets adults aged ≥45 years, a small number of younger participants were included due to its household-based sampling design. We excluded individuals aged <45 years to align with the original target population of CHARLS, reduce age-related heterogeneity, and remain consistent with previous CHARLS-based studies (24, 25). Of the 17,708 participants in the Wave 1 baseline, 10,234 individuals aged ≥45 years with complete baseline and follow-up data were included in the final analysis (**Figure 1**). The study was conducted in accordance with the Declaration of Helsinki and was approved by the Biomedical Ethics Review Committee of Peking University (IRB00001052-11015). All participants provided written informed consent.

**Figure 1:**
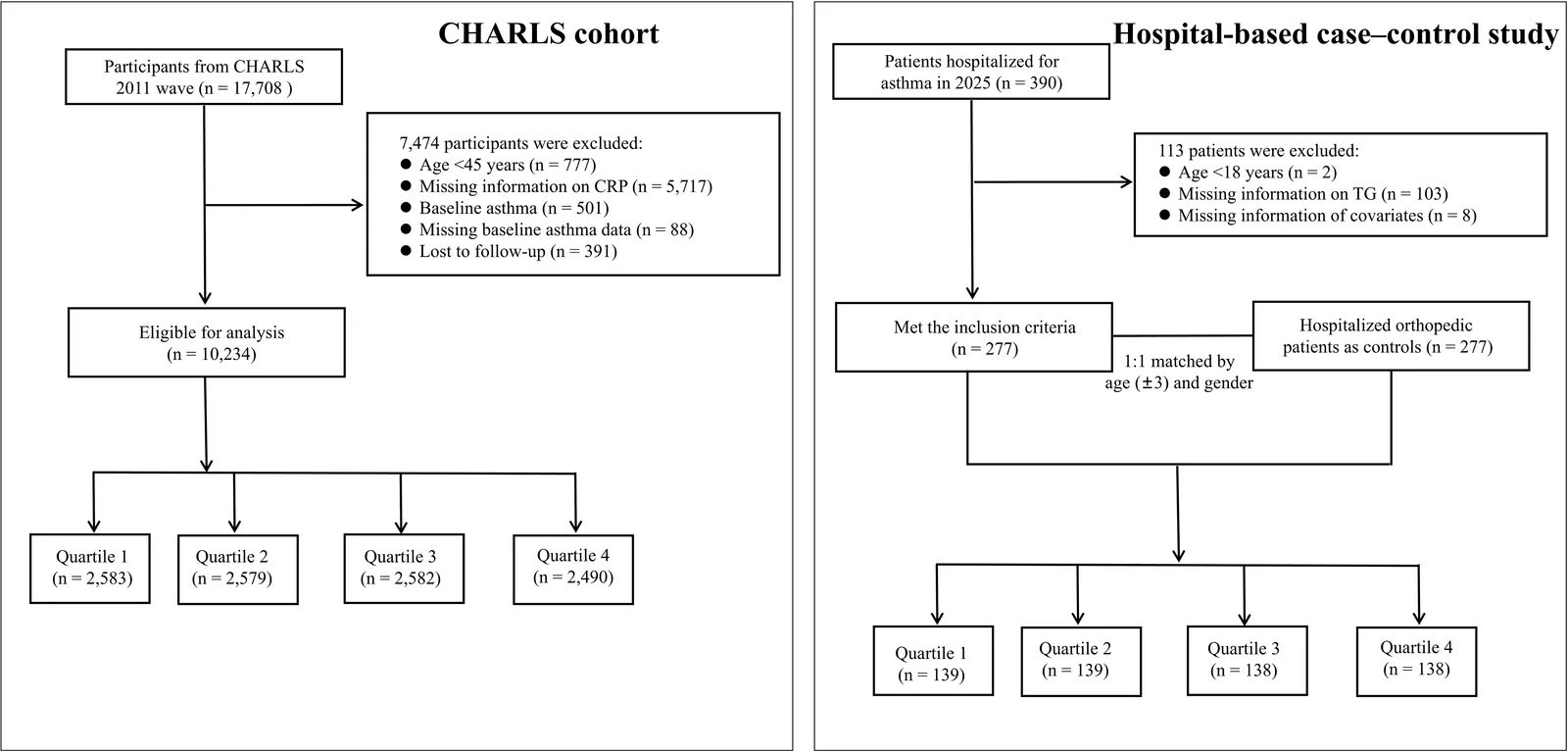
Flow chart of the study population.

An external validation dataset was obtained from hospitalized patients admitted to Wu Song Central Hospital (Baoshan District, Shanghai, China) between January 1 and December 31, 2025. Inclusion criteria were (1) hospitalized patients in 2025, with only the first record retained if multiple admissions existed; (2) age ≥18 years; (3) clinically diagnosed asthma; and (4) complete data for all indicators required to calculate CTI. Exclusion criteria were (1) patients aged <18 years; (2) presence of other respiratory diseases, including chronic obstructive pulmonary disease (COPD), chronic bronchitis, emphysema, bronchiectasis, and other chronic airway diseases; and (3) incomplete CTI exposure data or missing covariates. Based on the inclusion and exclusion criteria, a total of 277 hospitalized adults with asthma and 277 controls (1:1 matched by age ± 3 years and gender) from orthopedic hospitalized adults were included in the final analysis (**Figure 1**). Ethical approval for the validation population was obtained from the Clinical Trials Ethics Committee of Wu Song Central Hospital, Baoshan District, Shanghai (Approval No. 2026-P-02). The principles of the Helsinki Declaration were followed. All data involving medical records were not publicly available, and no participants could be identified from the study data.

### 2.2 Measurement of CTI and asthma

Lipid, glucose, and CRP levels were measured in blood samples obtained at CHARLS baseline and validation population. We calculated CTI using a formula from a prior publication (18):


CTI=0.412×ln(CRP [mg/L])+ln(TG [mg/dL]×FPG [mg/dL])/2

Where TG and FPG represent triglycerides and fasting plasma glucose, respectively.

In the CHARLS cohort, asthma was defined by participants’ self-report of a physician diagnosis (yes/no) and served as the primary outcome. Participants with a history of asthma at baseline (2011) were excluded, and incident cases were identified during follow-ups in 2013, 2015, and 2018. Follow-up time for each participant was calculated from the 2011 baseline to the earliest of incident asthma diagnosis, loss to follow-up, death, or completion of the 2018 survey.

In the validation population, asthma was clinically diagnosed by physicians during hospitalization based on a combination of patient-reported symptoms (such as recurrent wheezing, shortness of breath, chest tightness, and cough), medical history, and objective assessments including pulmonary function tests. Other respiratory diseases were excluded to ensure diagnostic specificity.

### 2.3 Assessments of covariates

Potential confounders were selected based on evidence from previous studies and the availability of relevant data (25–27). The covariates from CHARLS were sex, age, residence, education level, annual income, marital status, alcohol consumption, smoking status, physical activity, hypertension, diabetes, CVD, and body mass index (BMI). Sex (male or female) was self-reported, and age was included as a continuous variable. Residence was classified as urban or rural based on actual living location. Education level was categorized according to the Chinese system: no formal education, primary school (1–6 years), middle school (7–9 years), and high school or above (≥10 years). Annual income was divided into tertiles (T1–T3). Marital status was grouped as married, unmarried, or other (separated, divorced, or widowed). Smoking status and alcohol consumption were classified as never, former, or current. Physical activity was assessed using the CHARLS questionnaire, which asked participants whether they engaged in moderate or vigorous activities lasting at least 10 minutes at a time during the past week. Physical activity was categorized as none/light, moderate, or heavy. Diabetes was defined as fasting blood glucose ≥126 mg/dL (7.0 mmol/L), use of insulin or oral hypoglycemic agents, or a self-reported physician diagnosis (28). Hypertension was defined as systolic blood pressure ≥140 mmHg, diastolic blood pressure ≥90 mmHg, use of antihypertensive medication, or a self-reported diagnosis (28). Participants were considered to have CVD if they reported a history of stroke or heart disease. Body mass index (BMI) was calculated as weight in kilograms divided by height in meters squared (kg/m²).

Covariates in the validation population were selected to largely align with those used in CHARLS and were obtained through clinical record systems and physical measurements. Annual income and physical activity were not included in the analysis due to missing data. Diabetes, hypertension, and CVD were defined according to the definitions applied in CHARLS.

### 2.4 Statistical analysis

Continuous variables are expressed as mean ± standard deviation (SD), whereas categorical variables are summarized as counts and percentages. Baseline characteristics were compared between participants who developed asthma during follow-up and those who remained asthma-free using t-tests for continuous variables and chi-square tests for categorical variables. Differences between included and excluded participants were also evaluated. Using the Harmonized CHARLS database, most covariates had high completeness. Missing values of categorical covariates were handled using two predefined strategies. For variables with missing proportions <5%, including smoking status, alcohol consumption, and chronic disease status, missing values were assigned to the most frequent category. For variables with missing proportions ≥5%, including annual income and physical activity, missing values were treated as a separate category (“missing”) and included in the regression models. This approach allowed us to retain eligible participants while accounting for missing covariate information.

To inform covariate selection, a directed acyclic graph (DAG) was constructed to identify the minimal sufficient set of confounders (**Supplementary Figure 1**). The primary models were adjusted for age (continuous), residence, education level, annual income, marital status, smoking status, alcohol consumption, physical activity, and hypertension. Potential mediators, including hypertension, diabetes, CVD and/or BMI, were excluded from the main analyses to avoid overadjustment.

Cox proportional hazards regression models were used to examine the associations between CTI and incident asthma. Hazard ratios (HRs) with 95% confidence intervals (CIs) were estimated, with CTI analyzed both as a continuous variable and in quartiles, using the lowest quartile as the reference. The quartile cut-off values were defined as follows: Q1 (lowest quartile) ≤4.34, Q2 (second quartile) >4.34 to ≤4.71, Q3 (third quartile) >4.71 to ≤5.13, and Q4 (highest quartile) >5.13. Schoenfeld residuals were used to evaluate the proportional hazards assumption for CTI in all models, with no violations detected (all *P >*0.05). Three Cox regression models were constructed: Model 1 was unadjusted; Model 2 adjusted for age (continuous), residence, education, annual income, and marital status; and Model 3 additionally included smoking status, alcohol consumption, and physical activity. To investigate potential nonlinearity, we applied restricted cubic spline (RCS) models adjusting for Model 3 covariates. Knots were set at the 5th, 50th, and 95th percentiles of the CTI distribution, using the median as the reference point.

To investigate the stability of the CTI–asthma relationship and to identify vulnerable subgroups, we performed analyses stratified by demographic and lifestyle variables. The association between CTI and asthma was examined within each subgroup, and interaction terms were tested to identify effect modification. Four types of sensitivity analyses were conducted. First, we additionally adjusted for chronic diseases, BMI, or inflammatory biomarkers in Cox proportional hazards models. Second, we repeated the analyses after excluding participants with chronic lung diseases at baseline to minimize potential bias caused by coexisting respiratory diseases. Third, we assessed the robustness of the findings using an alternative CTI formula reported in previous studies (15, 18, 29): CTI = 0.412 × Ln (CRP [mg/L]) + Ln (TG [mg/dl] × FPG [mg/dl]/2). Fourth, we performed cross-sectional analyses using baseline data to examine the association between CTI and asthma prevalence.

Finally, conditional logistic regression models were applied to the validation dataset. Three models were constructed: Model 1 was unadjusted; Model 2 was adjusted for residence, education level, and marital status; and Model 3 was further adjusted for current smoking and current drinking. Additionally, RCS analyses were performed to assess potential dose–response relationships. Among adults with asthma, inhaled corticosteroid (ICS) use was further included as an additional covariate to account for its potential confounding effect. For analyses of blood inflammatory biomarkers (eosinophil and neutrophil counts), regression coefficients (β) were transformed into percentage changes using the formula: $(e^{\beta }-1)\times 100\%$.

All analyses were performed using R (version 4.1.3). Two-tailed *P* < 0.05 was considered statistically significant.

## 3 Results

### 3.1 Population characteristics

The analysis included 10,234 participants from the CHARLS cohort, with a median follow-up duration of 84 months. Among them, CTI values ranged from 3.07 to 7.53, with a mean of 4.76 ± 0.59. During this period, 436 new cases of asthma were identified, corresponding to an incidence of 4.26%. The mean age of the cohort was 58.75 ± 9.26 years, with 46.6% of participants being male and 36.2% living in urban areas. Baseline characteristics across CTI quartiles are summarized in **Table 1**. For instance, participants in higher CTI quartiles were generally older, more likely to live in urban areas, and less likely to be male. **Supplementary Table 1** also shows some differences in several characteristics between excluded (n = 7,474) and included (n = 10,234) participants.

**Table 1:** Baseline characteristics across CTI quartiles in CHARLS.

Characteristics		Overall (n = 10,234)	CTI quartiles (%)				*P* value
			Q1 (n = 2,583)	Q2 (n = 2,579)	Q3 (n = 2,582)	Q4 (n = 2,490)	
Sex	Male	4769 (46.6)	1238 (47.9)	1235 (47.9)	1172 (45.4)	1124 (45.1)	0.066
	Female	5465 (53.4)	1345 (52.1)	1344 (52.1)	1410 (54.6)	1366 (54.9)	
Age (years, continuous)		58.75 ± 9.26	57.32 ± 9.05	58.81 ± 9.20	59.39 ± 9.39	59.53 ± 9.22	<0.001
Age (years, categorical)	45–49	2054 (20.1)	668 (25.9)	506 (19.6)	467 (18.1)	413 (16.6)	<0.001
	50–59	3862 (37.7)	972 (37.6)	972 (37.7)	962 (37.3)	956 (38.4)	
	≥60	4318 (42.2)	943 (36.5)	1101 (42.7)	1153 (44.7)	1121 (45.0)	
Residence	Urban	3709 (36.2)	799 (30.9)	892 (34.6)	980 (38.0)	1038 (41.7)	<0.001
	Rural	6525 (63.8)	1784 (69.1)	1687 (65.4)	1602 (62.0)	1452 (58.3)	
Education level	No formal education	2947 (28.8)	757 (29.3)	743 (28.8)	710 (27.5)	737 (29.6)	0.326
	Primary school	4077 (39.8)	1016 (39.3)	1047 (40.6)	1067 (41.3)	947 (38.0)	
	Middle school	2082 (20.3)	528 (20.4)	504 (19.5)	539 (20.9)	511 (20.5)	
	High school or above	1128 (11.0)	282 (10.9)	285 (11.1)	266 (10.3)	295 (11.8)	
Annual income^a^	T1	2134 (20.9)	506 (19.6)	556 (21.6)	537 (20.8)	535 (21.5)	<0.001
	T2	2089 (20.4)	522 (20.2)	546 (21.2)	551 (21.3)	470 (18.9)	
	T3	2179 (21.3)	493 (19.1)	512 (19.9)	574 (22.2)	600 (24.1)	
	Missing	3832 (37.4)	1062 (41.1)	965 (37.4)	920 (35.6)	885 (35.5)	
Marital status	Married	9039 (88.3)	2309 (89.4)	2289 (88.8)	2266 (87.8)	2175 (87.3)	0.036
	Unmarried	79 (0.8)	28 (1.1)	16 (0.6)	17 (0.7)	18 (0.7)	
	Other	1116 (10.9)	246 (9.5)	274 (10.6)	299 (11.6)	297 (11.9)	
Smoking status	Never smoker	6458 (63.1)	1644 (63.6)	1606 (62.3)	1648 (63.8)	1560 (62.7)	0.011
	Former smoker	825 (8.1)	175 (6.8)	199 (7.7)	214 (8.3)	237 (9.5)	
	Current smoker	2951 (28.8)	764 (29.6)	774 (30.0)	720 (27.9)	693 (27.8)	
Alcohol consumption	Never drinker	6016 (58.8)	1460 (56.5)	1487 (57.7)	1561 (60.5)	1508 (60.6)	<0.001
	Former drinker	824 (8.1)	186 (7.2)	206 (8.0)	218 (8.4)	214 (8.6)	
	Current drinker	3394 (33.2)	937 (36.3)	886 (34.4)	803 (31.1)	768 (30.8)	
Physical activity	None/Light	1438 (14.1)	301 (11.7)	329 (12.8)	370 (14.3)	438 (17.6)	<0.001
	Moderate	1273 (12.4)	290 (11.2)	321 (12.4)	347 (13.4)	315 (12.7)	
	Heavy	1539 (15.0)	497 (19.2)	410 (15.9)	347 (13.4)	285 (11.4)	
	Missing	5984 (58.5)	1495 (57.9)	1519 (58.9)	1518 (58.8)	1452 (58.3)	
Hypertension	No	6146 (60.1)	1859 (72.0)	1663 (64.5)	1440 (55.8)	1184 (47.6)	<0.001
	Yes	4088 (39.9)	724 (28.0)	916 (35.5)	1142 (44.2)	1306 (52.4)	
Diabetes	No	8551 (83.6)	2445 (94.7)	2313 (89.7)	2174 (84.2)	1619 (65.0)	<0.001
	Yes	1683 (16.4)	138 (5.3)	266 (10.3)	408 (15.8)	871 (35.0)	
Cardiovascular disease	No	8815 (86.1)	2336 (90.4)	2258 (87.6)	2205 (85.4)	2016 (81.0)	<0.001
	Yes	1419 (13.9)	247 (9.6)	321 (12.4)	377 (14.6)	474 (19.0)	
BMI (kg/m^2^)	23.43 ± 3.54	22.08 ± 2.96	22.90 ± 3.28	24.01 ± 3.47	24.84 ± 3.81	<0.001	

CTI, C-reactive protein-triglyceride-glucose index; Q1, first quartile; Q2, second quartile; Q3, third quartile; Q4, fourth quartile.

^a^ Annual income tertiles were categorized as follows: T1 (lowest tertile), T2 (middle tertile), and T3 (highest tertile).

### 3.2 Associations between CTI and asthma incidence

The association between CTI and the risk of incident asthma was evaluated using Cox proportional hazards models (**Table 2**). When treated as a continuous variable, CTI was consistently associated with an increased risk of asthma across all models. For instance, in the fully adjusted model (Model 3), per 1-unit increase in CTI was associated with a 24% higher risk of asthma (HR = 1.24, 95% CI: 1.06–1.45). Compared with the lowest quartile (Q1), participants in the second, third, and highest quartiles (Q2–Q4) exhibited progressively higher risks of asthma (HRs = 1.08, 1.13, and 1.33, respectively; *P* for trend = 0.035).

**Table 2:** Association of the CTI with asthma incidence using Cox proportional hazards models from CHARLS.

Characteristics	Model 1		Model 2		Model 3	
	HR (95% CI)	*P* value	HR (95% CI)	*P* value	HR (95% CI)	*P* value
CTI (per 1 unit)	1.25 (1.07, 1.46)	0.004	1.25 (1.07, 1.47)	0.004	1.24 (1.06, 1.45)	0.008
CTI quartile						
Q1 (≤4.34)	Reference		Reference		Reference	
Q2 (>4.34 to ≤4.71)	1.11 (0.84, 1.47)	0.446	1.09 (0.83, 1.44)	0.549	1.08 (0.82, 1.43)	0.580
Q3 (>4.71 to ≤5.13)	1.18 (0.90, 1.55)	0.235	1.15 (0.87, 1.51)	0.319	1.13 (0.86, 1.49)	0.367
Q4 (>5.13)	1.38 (1.05, 1.80)	0.019	1.37 (1.05, 1.79)	0.022	1.33 (1.02, 1.75)	0.037
*P* for trend		0.018		0.021		0.035

CTI, C-reactive protein-triglyceride-glucose index; HR, hazard ratio; CI, confidence interval; Q1, first quartile; Q2, second quartile; Q3, third quartile; Q4, fourth quartile.

Model 1: unadjusted.

Model 2: adjusted for sex, age (continuous), residence, education level, annual income, and marital status.

Model 3: additionally adjusted for smoking status, alcohol consumption, and physical activity.

The association between CTI and incident asthma risk, modeled using RCS, is presented in **Figure 2**. The risk of asthma increased progressively with higher CTI levels (overall *P* = 0.032), and no significant departure from linearity was observed (*P* for non-linearity = 0.376), suggesting an approximately linear association.

**Figure 2:**
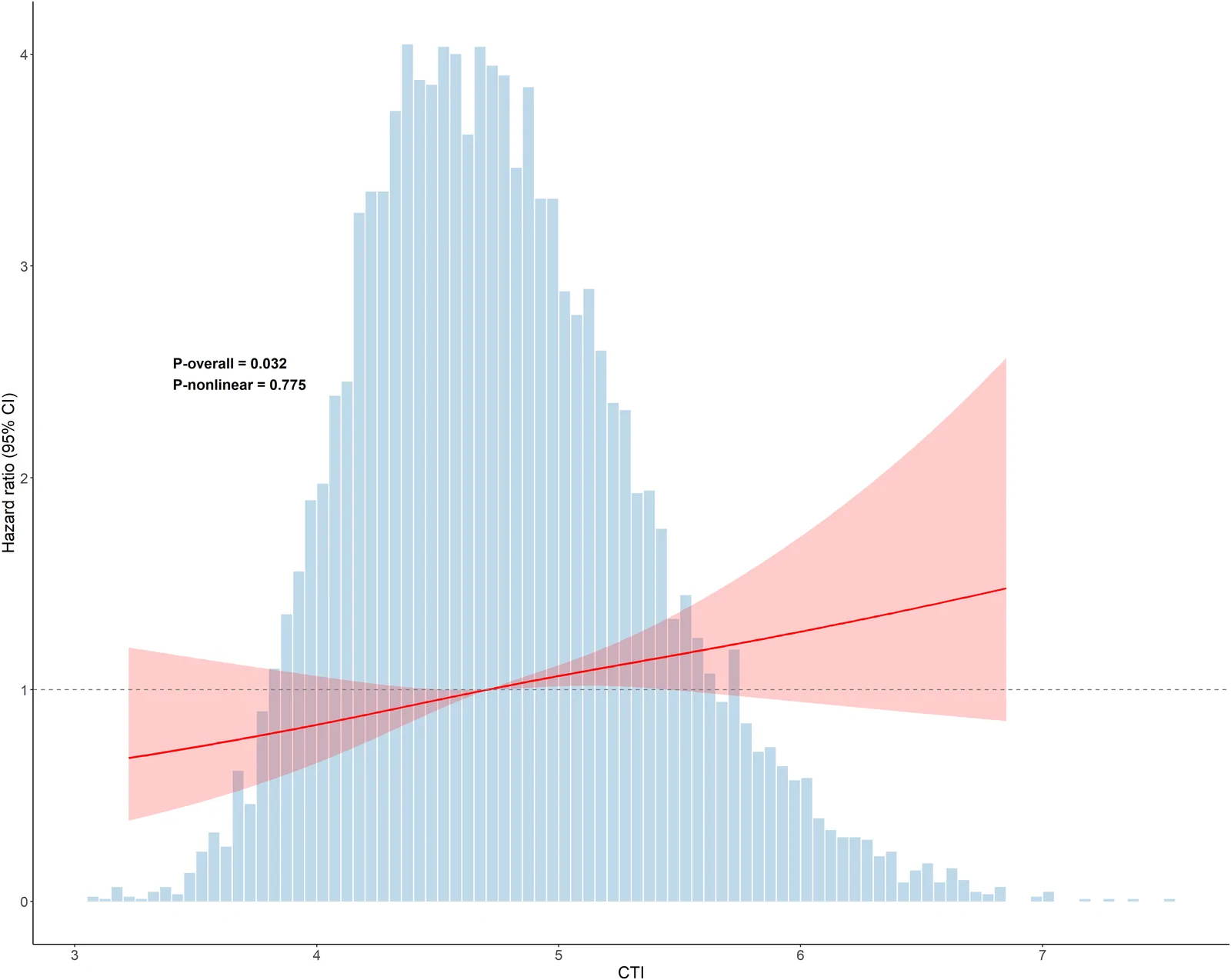
Dose–response relationship of CTI with asthma incidence in CHARLS. Knots were set at the 5th, 50th, and 95th percentiles of the CTI distribution. The median value is used as the reference. Adjusted for sex, age (continuous), residence, education level, annual income, and marital status, smoking status, alcohol consumption, and physical activity.

### 3.3 Stratified and sensitivity analyses

We performed subgroup analyses to examine the stability of the association between CTI and asthma incidence and to explore potentially vulnerable populations (**Figure 3**). Overall, the positive association persisted across most subgroups. Stronger effects were observed in participants aged 45–49 years, males, unmarried individuals, and those who reported never drinking alcohol. These differences were supported by statistically significant interaction tests.

**Figure 3:**
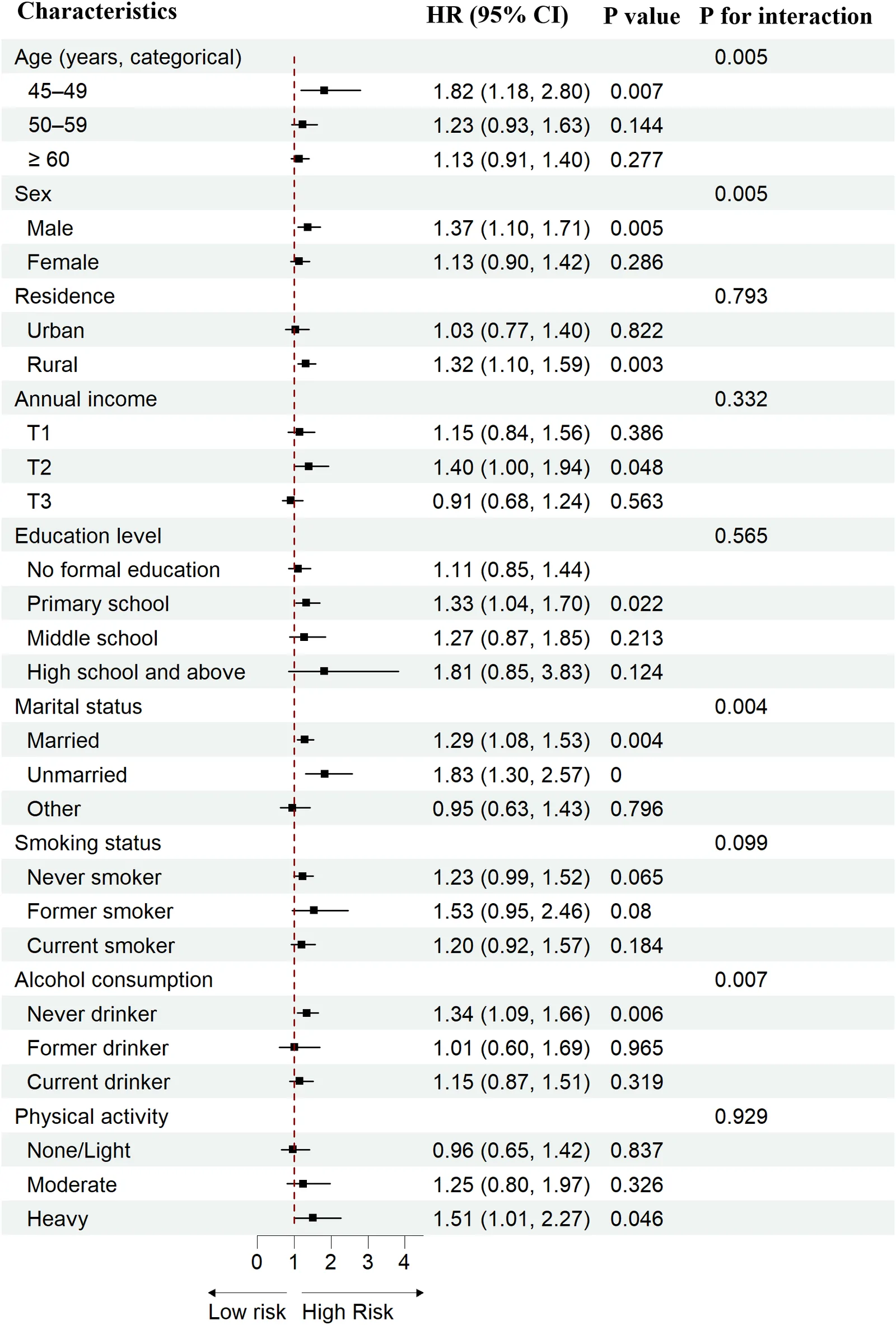
Subgroup analysis for the association between CTI and asthma incidence. Adjusted for sex, age (continuous), residence, education level, annual income, and marital status, smoking status, alcohol consumption, and physical activity.

In sensitivity analyses further adjusting for chronic diseases, BMI, or inflammatory biomarkers, the association between CTI and asthma remained robust (**Supplementary Tables 2**, **S3**). Specifically, the HRs per 1-unit increase in CTI were 1.22 (95% CI: 1.03–1.44) with adjustment for chronic diseases and 1.21 (95% CI: 1.03–1.42) with adjustment for inflammatory biomarkers. After excluding participants with chronic lung diseases at baseline, the association between CTI and asthma incidence remained largely unchanged, with per 1-unit increase in CTI associated with a higher risk of asthma incidence (HR = 1.21, 95% CI: 1.00–1.47) (**Supplementary Table 4**). Sensitivity analysis using an alternative CTI formula yielded results consistent with those of the main analysis, further supporting the robustness of the findings (**Supplementary Table 5**; **Supplementary Figure 2**). In addition, cross-sectional analyses at baseline demonstrated positive associations between CTI and asthma prevalence (**Supplementary Table 6**; **Supplementary Figure 3**), with findings consistent with the longitudinal analyses.

### 3.4 External validation

**Supplementary Table 7** presents the baseline characteristics of the external validation population across CTI levels. For instance, participants with higher CTI values were more likely to be male, current smokers, and diabetes. No significant differences were observed for age, residence, education level, marital status, current drinking, hypertension, or CVD. **Supplementary Table 8** further compares the baseline characteristics between participants with and without asthma after age- and sex-matching. The results indicated that age and sex were comparable between the two groups. Only residence, current smoking status, and hypertension remained significantly different between the asthma and non-asthma groups, whereas no significant differences were observed for the other covariates.

Conditional logistic regression in the external validation population showed results consistent with the CHARLS cohort (**Table 3**). For example, in the fully adjusted model, when CTI was treated as a continuous variable, per unit increase in CTI was associated with higher odds of asthma (OR = 1.31, 95% CI: 1.06–1.61). RCS analysis revealed an approximately inverted U-shaped association between CTI and asthma, which remained similar after further adjustment for chronic diseases (**Figure 4**; **Supplementary Figure 4**). Differences in curve shape between the CHARLS cohort and the validation population, particularly greater variability at the tails, may partly reflect the small validation sample size.

**Table 3:** Association of the CTI with asthma using conditional logistic regression models from external validation population.

Characteristics	Model 1		Model 2		Model 3	
	OR (95% CI)	*P* value	OR (95% CI)	*P* value	OR (95% CI)	*P* value
CTI (per 1 unit)	1.28 (1.05, 1.55)	0.015	1.33 (1.08, 1.62)	0.006	1.31 (1.06, 1.61)	0.012
CTI quartile						
Q1 (≤4.17)	Reference		Reference		Reference	
Q2 (>4.17 to ≤4.83)	1.53 (0.98, 2.40)	0.062	1.50 (0.95, 2.37)	0.085	1.45 (0.91, 2.32)	0.118
Q3 (>4.83 to ≤5.61)	1.68 (1.06, 2.65)	0.027	1.70 (1.07, 2.73)	0.026	1.65 (1.02, 2.66)	0.040
Q4 (>5.61)	1.93 (1.18, 3.14)	0.008	2.07 (1.26, 3.42)	0.004	1.96 (1.17, 3.28)	0.010
*P* for trend		0.008		0.004		0.008

CTI, C-reactive protein-triglyceride-glucose index; OR, odds ratio; CI, confidence interval; Q1, first quartile; Q2, second quartile; Q3, third quartile; Q4, fourth quartile.

Model 1: unadjusted.

Model 2: adjusted for residence, education level, and marital status.

Model 3: additionally adjusted for current smoking and current drinking.

**Figure 4:**
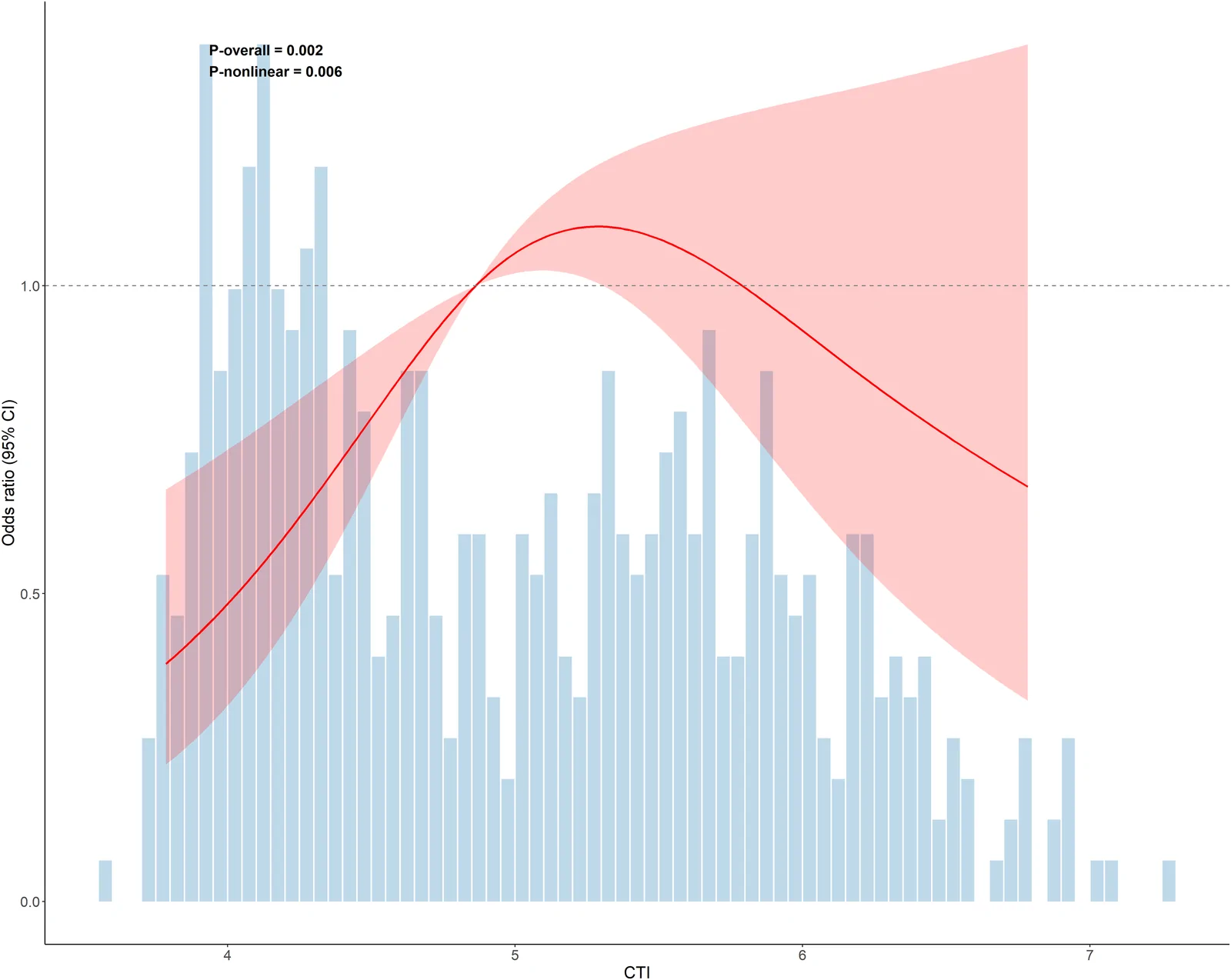
Dose–Response relationship of CTI with asthma in the external validation population. Knots were set at the 5th, 50th, and 95th percentiles of the CTI distribution. The median value is used as the reference. Adjusted for residence, education level, marital status, current smoking, and current drinking.

Higher CTI levels were consistently associated with increased eosinophil and neutrophil counts among adults with asthma (**Table 4**). In the fully adjusted model, per 1-unit increase in CTI was associated with an 8.00% increase in eosinophil counts (95% CI: 4.58%–11.53%) and a 16.72% increase in neutrophil counts (95% CI: 8.24%–25.86%). Compared with participants in the Q1, those in the Q4 exhibited significantly higher eosinophil counts (17.46% increase; 95% CI: 9.08%–26.49%) and neutrophil counts (40.41% increase; 95% CI: 18.14%–66.87%). Eosinophil and neutrophil counts increased progressively across CTI quartiles, with significant linear trends observed for both biomarkers (both *P* for trend <0.001).

**Table 4:** Associations of the CTI with blood eosinophil and neutrophil counts among adults with asthma in an external validation population^a^.

Characteristics	Eosinophils % change (95% CI)	*P* value	Neutrophils % change (95% CI)	*P* value
CTI (per 1 unit)	8.00 (4.58, 11.53)	<0.001	16.72 (8.24, 25.86)	<0.001
CTI quartile				
Q1 (≤4.17)	Reference		Reference	
Q2 (>4.17 to ≤4.83)	5.58 (-1.92, 13.65)	0.148	12.18 (-5.53, 33.21)	0.189
Q3 (>4.83 to ≤5.61)	6.32 (-1.26, 14.47)	0.104	12.33 (-5.46, 33.47)	0.185
Q4 (>5.61)	17.46 (9.08, 26.49)	<0.001	40.41 (18.14, 66.87)	<0.001
*P* for trend		<0.001		<0.001

CTI, C-reactive protein-triglyceride-glucose index; CI, confidence interval; Q1, first quartile; Q2, second quartile; Q3, third quartile; Q4, fourth quartile.

^a^ Model was adjusted for residence, education level, marital status, current smoking status, current drinking status, and inhaled corticosteroid (ICS) use.

Overall, the external validation population largely confirmed the main findings from CHARLS. Higher CTI levels were associated with increased asthma risk and elevated inflammatory biomarkers among adults with asthma, suggesting that CTI may contribute to asthma development and inflammatory responses.

## 4 Discussion

### 4.1 Key findings

In the longitudinal study, higher CTI was associated with increased asthma risk, with RCS analyses revealing a predominantly linear dose–response relationship. For instance, a 1-unit increase in CTI corresponded to a 24% higher risk of asthma (HR = 1.24, 95% CI: 1.06–1.45). Subgroup analyses showed stronger associations in participants aged 45–49 years, males, unmarried individuals, and never drinkers, highlighting potentially vulnerable populations. Importantly, these findings were generally supported by the hospital-based validation population, demonstrating the robustness and consistency of the association between CTI and asthma across different populations. Moreover, among adults with established asthma, higher CTI levels were associated with increased eosinophil and neutrophil counts, suggesting that elevated CTI may also reflect enhanced inflammatory responses in individuals with asthma. Collectively, our findings suggest that CTI may serve as a valuable biomarker for identifying individuals at higher risk of asthma and characterizing asthma-related inflammatory profiles, with potential implications for early risk stratification and targeted preventive interventions.

### 4.2 Comparison with previous studies and potential explanations

The CTI combines markers of systemic inflammation and IR, and has increasingly been adopted as a clinical research tool (18). In a cohort of 10,443 Chinese adults from CHARLS, higher CTI was significantly associated with an increased risk of stroke, with HR of 1.22 (95% CI: 1.09–1.36) in females and 1.15 (95% CI: 1.02–1.29) in males (19). Similarly, in a cohort of 8,679 US adults, higher CTI was strongly associated with increased risk of CVD mortality (HR = 2.28, 95% CI: 1.69–3.24) and all-cause mortality (HR = 2.14, 95% CI: 1.76–2.55) (20). To date, the vast majority of studies have focused on cardiovascular outcomes, with only a few exploring cognitive decline, non-alcoholic fatty liver disease, and cancer (30–32). However, evidence regarding the relationship between CTI and respiratory outcomes, particularly asthma, remains limited. To date, only two recent studies have explored this association using cross-sectional data (23, 24). One study based on the National Health and Nutrition Examination Survey (NHANES) and the China Health and Nutrition Survey (CHNS) reported a positive association between CTI and asthma prevalence, while another study using dual national cohorts similarly observed an association between CTI and asthma prevalence (23, 24). However, these studies were limited by their cross-sectional designs, lack of external validation using independent clinical data, and limited evaluation of asthma-related inflammatory biomarkers. To address these knowledge gaps, we found that higher CTI levels were associated with an increased risk of asthma, with this association further supported by an independent hospital-based validation dataset. Furthermore, among adults with asthma, per 1-unit increase in CTI was associated with an 8.00% increase in eosinophil counts (95% CI: 4.58%–11.53%) and a 16.72% increase in neutrophil counts (95% CI: 8.24%–25.86%). By demonstrating the associations of CTI with asthma-related inflammatory biomarkers, our study provides additional insights into the potential biological pathways linking metabolic–inflammatory dysregulation with asthma.

Based on the definition of CTI, we reviewed previous studies examining the associations of its main components (CRP and TyG) with asthma. CRP, a commonly measured and relatively stable marker of systemic inflammation, has been linked to asthma in previous studies (10, 11). In a study of 281 asthmatic patients and 177 controls, higher CRP levels were associated with increased risk of severe asthma, with adjusted ORs of 3.49 (95% CI: 1.51–8.12) and 6.46 (95% CI: 2.85–16.62) for the third and fourth quartiles, respectively (10). A two-sample Mendelian randomization study reported that higher genetically predicted CRP levels increase the risk of asthma, especially obesity-related asthma (33). The positive association between CRP levels and asthma is biologically plausible, as CRP reflects systemic low-grade inflammation that can contribute to airway inflammation and hyperresponsiveness (34). Specifically, elevated CRP has been shown to promote pro-inflammatory cytokine release, enhance recruitment of immune cells to the airways, and exacerbate airway remodeling, all of which can increase asthma risk and severity (35, 36).

The TyG index is a widely used surrogate marker of IR in clinical and epidemiological studies (15). Several recent studies have reported that elevated TyG levels are associated with an increased risk of asthma (16, 17). In an NHANES analysis of 1,629 US adolescents, higher TyG levels were associated with increased asthma prevalence, with those in the highest quintile showing significantly higher odds of asthma than those in the reference group (adjusted OR = 4.26, 95% CI: 1.54–11.81) (16). Beyond asthma occurrence, emerging evidence suggested that higher TyG levels were also linked to greater disease severity among individuals with asthma (37, 38). For instance, Wen et al. reported a linear association between higher TyG levels and increased blood eosinophil counts, with each 1-unit increment in TyG associated with an approximately 12.4 cells/µL increase in eosinophil counts after full adjustment (37). Elevated TyG reflects IR and metabolic dysregulation, which may promote asthma development through airway hyperresponsiveness, remodeling, and systemic inflammation, providing potential mechanistic insights into the observed association between CTI and asthma (39).

Taken together, previous studies on CRP and TyG provide supportive evidence for our findings linking higher CTI levels to increased asthma risk and suggest potential biological mechanisms underlying this association. Moreover, a recent study on the joint effect of TyG and CRP levels on chronic obstructive pulmonary disease supported the potential of integrating these markers into a composite index (40). Furthermore, our external validation analysis revealed that higher CTI levels were associated with increased eosinophil and neutrophil counts among adults with asthma, providing additional evidence that CTI may be linked to systemic inflammatory responses underlying asthma development and progression. These findings provide first-hand evidence supporting future investigations into the biological pathways linking metabolic inflammation and asthma, as well as the development of preventive and therapeutic strategies.

### 4.3 Subgroup analysis

We conducted comprehensive subgroup analyses to evaluate the association between CTI and asthma risk across different demographic and lifestyle characteristics. The association was stronger among participants aged 45–49 years, males, unmarried individuals, and never drinkers, with statistically significant interactions observed (*P* < 0.05), indicating that these groups may be particularly susceptible to the effects of elevated CTI. Although no previous studies have directly examined age-specific differences in the association between CTI and asthma, adult cohort data indicate that systemic inflammation and metabolic dysregulation tend to be more pronounced in middle-aged populations (41). Specifically, studies in cardiometabolic research have demonstrated that the associations of CTI with disease outcomes are often amplified during midlife, suggesting that metabolic–inflammatory disturbances may have greater clinical relevance during this period (41). This may partly explain the stronger CTI–asthma association observed in participants aged 45–49 years in our study.

Sex differences in inflammatory and metabolic profiles may partly explain the stronger CTI–asthma association observed among males (42, 43). Differences in adipose tissue distribution, body composition, and metabolic regulation between males and females may influence systemic inflammatory markers, including CRP and IL-6, thereby contributing to sex-specific inflammatory profiles (42). In addition, differences in hormonal regulation may provide another potential explanation for the observed sex-specific associations. Sex hormones play important roles in regulating asthma pathogenesis by modulating immune responses and inflammatory pathways; however, the mechanisms through which sex hormones initiate and drive inflammatory responses in asthma remain incompletely understood (44). These biological differences in metabolic regulation, immune function, and hormonal signaling may contribute to variations in asthma susceptibility associated with systemic inflammation–metabolism disturbances. Nevertheless, further studies incorporating detailed measurements of sex hormones, immune profiles, and metabolic characteristics are needed to directly elucidate the mechanisms underlying these sex-specific effects.

The stronger association observed among unmarried individuals may partly reflect differences in psychosocial stress, social support, and health-related behaviors (45). Previous studies have suggested that reduced social support and chronic psychosocial stress may activate neuroendocrine and inflammatory pathways, contributing to elevated systemic inflammation (46–49). Specifically, longitudinal evidence from the Midlife Development in the United States study demonstrated that perceived social support from family, friends, and spouses was modestly associated with lower levels of systemic inflammation (49). These findings suggest that social relationships may influence immune regulation and inflammatory processes, providing a potential pathway linking marital status with differences in inflammation-related disease susceptibility.

The stronger association among never drinkers may reflect differences in underlying health status, lifestyle patterns, or residual confounding (50). Previous studies have suggested that moderate alcohol consumption may be associated with lower levels of systemic inflammatory markers, which may provide another possible explanation for the weaker association observed among drinkers (51, 52). In the CHARLS population, alcohol consumption was categorized as never, former, and current drinking, without further classification according to drinking quantity. Therefore, we could not directly assess whether the observed association differed according to drinking intensity. Nevertheless, given that CHARLS mainly includes middle-aged and older adults, heavy drinking may represent a relatively smaller proportion of alcohol consumption patterns in this population. However, this finding should not be interpreted as supporting alcohol consumption for anti-inflammatory or health-promoting purposes, given the well-established adverse effects of alcohol use.

### 4.4 Public health and clinical implications

Our findings may have important implications for population-based asthma risk stratification and targeted prevention. Although asthma has traditionally been viewed as an airway-centered inflammatory disease, accumulating evidence suggests that systemic inflammation and metabolic dysfunction contribute to asthma susceptibility and disease heterogeneity (53, 54). In this study, CTI, a composite indicator integrating inflammatory and metabolic components, was prospectively associated with incident asthma risk. External validation further showed that higher CTI levels were associated with elevated eosinophil and neutrophil counts, supporting CTI as a potential marker of an inflammation–metabolism phenotype related to asthma susceptibility. CTI may serve as a feasible and scalable marker for identifying individuals at increased risk of asthma, as it is derived from routinely available clinical measurements. Individuals with elevated CTI levels may represent a subgroup characterized by concomitant systemic inflammation and metabolic dysfunction, suggesting that future asthma prevention and management strategies may benefit from considering both inflammatory phenotypes and metabolic status. Therapeutic strategies integrating anti-inflammatory treatments with interventions targeting metabolic abnormalities are currently being explored, particularly in obesity-related asthma phenotypes (12, 55). Our findings extend this concept by suggesting that the interplay between inflammation and metabolism may also be relevant to asthma susceptibility in other populations. Future studies are warranted to determine whether incorporating CTI into existing risk prediction models improves asthma risk discrimination and whether interventions targeting CTI-related inflammatory and metabolic abnormalities can reduce asthma risk.

### 4.5 Strengths and limitations

The study has several strengths. First, this is the first study to investigate the longitudinal association between CTI and incident asthma risk in a large, population-based cohort, extending previous cross-sectional evidence. Second, the robustness of our findings was further supported by comprehensive subgroup analyses and external validation data, which enhanced the reliability and generalizability of the results. Third, beyond identifying the association between CTI and asthma risk, our external validation analysis among adults with asthma further demonstrated that higher CTI levels were associated with increased eosinophil and neutrophil counts, providing additional insights into the potential inflammatory mechanisms linking metabolic dysregulation to asthma.

Several limitations of this study should be acknowledged. First, although the CHARLS population is a nationally representative sample of middle-aged and older adults in China, the generalizability of our findings to populations with different demographic or clinical characteristics, particularly younger individuals, should be interpreted with caution. Nevertheless, the external validation population, consisting of clinically identified asthma patients and matched individuals who were all aged ≥18 years, provided supportive evidence for the robustness of our findings and suggested broader applicability of the observed associations beyond the middle-aged and older population represented in CHARLS. Second, the case-control design of the external validation population limited the assessment of temporal relationships and causal inference. Future prospective studies using independent longitudinal cohorts are warranted to further validate our findings and assess the consistency of the observed associations across different populations. Third, asthma ascertainment differed between the CHARLS and external validation populations. In CHARLS, asthma was identified based on self-reported physician-diagnosed information, whereas the external validation population used clinical diagnoses based on respiratory symptoms, medical history, and objective assessments including pulmonary function tests. The reliance on self-reported asthma diagnosis in CHARLS may introduce potential outcome misclassification. Such misclassification, if non-differential with respect to CTI levels, could bias the associations toward the null and attenuate the observed effects. Moreover, differences in asthma ascertainment methods between the two populations may reduce comparability and potentially weaken the consistency of findings across datasets. Nevertheless, the consistent associations observed despite different diagnostic approaches further support the robustness and stability of our findings. Fourth, asthma-related inflammatory biomarkers were limited in both the primary and validation datasets. In the CHARLS cohort, inflammatory biomarkers such as eosinophil and neutrophil counts, FeNO, and immunoglobulin E (IgE) were not collected. In the validation dataset, although eosinophil and neutrophil counts were available, FeNO data could not be accessed because they were only recorded in original paper-based medical records and were unavailable in the electronic database. Moreover, IgE measurements were available for only approximately 30% of participants and were therefore not included in the analysis. Future studies incorporating more comprehensive asthma-related biomarkers are warranted to further elucidate the underlying mechanisms. Finally, although we adjusted for numerous potential confounders, including chronic diseases and BMI in sensitivity analyses, some unmeasured or incompletely characterized factors may still exist. For example, information on family history of asthma was not available in the current study, and residual confounding cannot be entirely excluded. Previous studies have suggested that genetic factors contribute substantially to asthma susceptibility (56). Future studies integrating genetic information, such as asthma-related genetic variants or polygenic risk scores, are warranted to further investigate whether genetic susceptibility modifies the association between CTI and asthma risk.

## 5 Conclusion

This study is the first to demonstrate that CTI is positively associated with incident asthma risk in a large adult population, with the findings consistently supported by external validation data. Stronger associations were observed among middle-aged adults, males, unmarried individuals, and never drinkers. Importantly, external validation among adults with asthma further showed that higher CTI levels were associated with elevated eosinophil and neutrophil counts, providing additional evidence that metabolic–inflammatory dysregulation may contribute to asthma pathogenesis. These findings provide robust evidence supporting the potential utility of CTI as a composite biomarker for asthma risk stratification. Future studies are warranted to further elucidate the biological mechanisms underlying the association between CTI and asthma development.

## Data Availability

The original contributions presented in the study are included in the article/**Supplementary Material**. Further inquiries can be directed to the corresponding author.
