# Supplementary material for: Associations between C-reactive protein-triglyceride glucose index and asthma risk among Chinese adults: evidence from the CHARLS cohort and external hospital-based validation
Source: Front Immunol. 2026 Sep 28;17:1837567. doi: 10.3389/fimmu.2026.1837567 (PMC13654970; doi:10.3389/fimmu.2026.1837567)
Supplement: Supplementary file 1 [file SupplementaryFile1.docx]

**Associations between C-reactive protein-triglyceride glucose index and asthma risk among Chinese adults: Evidence from the CHARLS cohort and external hospital-based validation**

**Contents**

**Fig. S1.** Directed acyclic graph of the relationship between the CTI and asthma.

**Fig. S2.** Dose–Response relationship of CTI with asthma incidence in CHARLS based on the different definition.

**Fig. S3.** Dose–Response relationship of CTI with asthma in CHARLS: A cross-sectional analysis.

**Fig. S4** Dose–Response relationship of CTI with asthma in the external validation population. adjusted for chronic diseases.

**Table S1** Comparison of characteristics between included and excluded participants in the CHARLS cohort.

**Table S2.** Association of the CTI with asthma incidence using Cox proportional hazards models with additional adjustment for chronic diseases or BMI.

**Table S3.** Association of the CTI with asthma incidence using Cox proportional hazards models adjusted for inflammatory biomarkers.

**Table S4.** Association of the CTI with asthma incidence using Cox proportional hazards models after excluding participants with chronic lung diseases at baseline.

**Table S5.** Association of the CTI with asthma incidence using Cox proportional hazards models using an alternative formula.

**Table S6.** Association between the CTI and asthma prevalence using logistic regression models.

**Table S7.** Baseline characteristics across CTI quartiles in external validation population.

**Table S8.** Baseline characteristics across asthma in external validation population.

**Fig. S1.** Directed acyclic graph of the relationship between the CTI and asthma.

**Fig. S2.** **Dose–Response relationship of CTI with asthma incidence in CHARLS** **based on the different definition**. Note: Knots were set at the 5th, 50th, and 95th percentiles of the CTI distribution. The median value is used as the reference. Adjusted for sex, age (continuous), residence, education level, annual income, and marital status, smoking status, alcohol consumption, and physical activity.

**Fig. S3.** **Dose–Response relationship of CTI with asthma in CHARLS: A cross-sectional analysis.** Note: Knots were set at the 5th, 50th, and 95th percentiles of the CTI distribution. The median value is used as the reference. Adjusted for sex, age (continuous), residence, education level, annual income, and marital status, smoking status, alcohol consumption, and physical activity.

 **Fig. S4** **Dose–Response relationship of CTI with asthma in the external validation population** **adjusted for** **chronic diseases.** Note: Knots were set at the 5th, 50th, and 95th percentiles of the CTI distribution. The median value is used as the reference. Adjusted for residence, education level, marital status, current smoking, current drinking, hypertension, diabetes, and cardiovascular disease.

**Table S1** Comparison of characteristics between included and excluded participants in the CHARLS cohort

| Characteristics | | Excluded participants | Included participants | *P* value |
| --- | --- | --- | --- | --- |
|  |  | (n = 7,474)^a^ | (n = 10,234) |  |
| Sex | Male | 3709 (49.6) | 4769 (46.6) | <0.001 |
|  | Female | 3763 (50.4) | 5465 (53.4) |  |
| Age (years, continuous) |  | 58.13 (11.32) | 58.75 (9.26) | <0.001 |
| Age (years, categorical) | 45–49 | 2013 (27.6) | 2054 (20.1) | <0.001 |
|  | 50–59 | 2314 (31.7) | 3862 (37.7) |  |
|  | ≥60 | 2972 (40.7) | 4318 (42.2) |  |
| Residence | Urban | 3462 (46.3) | 3709 (36.2) | <0.001 |
|  | Rural | 4012 (53.7) | 6525 (63.8) |  |
| Education level | No formal education | 1904 (25.5) | 2947 (28.8) | <0.001 |
|  | Primary school | 2843 (38.1) | 4077 (39.8) |  |
|  | Middle school | 1595 (21.4) | 2082 (20.3) |  |
|  | High school and above | 1116 (15.0) | 1128 (11.0) |  |
| Annual income^b^ | T1 | 1309 (17.5) | 2134 (20.9) | <0.001 |
|  | T2 | 1394 (18.7) | 2089 (20.4) |  |
|  | T3 | 1436 (19.2) | 2179 (21.3) |  |
|  | Missing | 3335 (44.6) | 3832 (37.4) |  |
| Marital status | Married | 6378 (85.3) | 9039 (88.3) | <0.001 |
|  | Unmarried | 80 (1.1) | 79 (0.8) |  |
|  | Other | 1016 (13.6) | 1116 (10.9) |  |
| Smoking status | Never smoker | 4962 (66.4) | 6458 (63.1) | <0.001 |
|  | Former smoker | 592 (7.9) | 825 (8.1) |  |
|  | Current smoker | 1920 (25.7) | 2951 (28.8) |  |
| Alcohol consumption | Never drinker | 4482 (60.0) | 6016 (58.8) | 0.139 |
|  | Former drinker | 619 (8.3) | 824 (8.1) |  |
|  | Current drinker | 2373 (31.8) | 3394 (33.2) |  |
| Physical activity | None/Light | 1046 (14.0) | 1438 (14.1) | <0.001 |
|  | Moderate | 794 (10.6) | 1273 (12.4) |  |
|  | Heavy | 822 (11.0) | 1539 (15.0) |  |
|  | Missing | 4812 (64.4) | 5984 (58.5) |  |
| Hypertension | No | 4797 (64.2) | 6146 (60.1) | <0.001 |
|  | Yes | 2677 (35.8) | 4088 (39.9) |  |
| Diabetes | No | 6860 (91.8) | 8551 (83.6) | <0.001 |
|  | Yes | 614 (8.2) | 1683 (16.4) |  |
| Cardiovascular disease | No | 6391 (85.5) | 8815 (86.1) | 0.247 |
|  | Yes | 1083 (14.5) | 1419 (13.9) |  |

^a^: Numbers may not add up to 7,474 due to missing data.

^b^: Annual income tertiles were categorized as follows: T1 (lowest tertile), T2 (middle tertile), and T3 (highest tertile).

**Table S2.** Association of the CTI with asthma incidence using Cox proportional hazards models with additional adjustment for chronic diseases or BMI

| Characteristics |  | HR (95% CI) | *P* value |
| --- | --- | --- | --- |
| Adjusted for chronic diseases^a^ |  |  |  |
| CTI (per 1 unit) |  | 1.22 (1.03, 1.44) | 0.021 |
| CTI quartile |  |  |  |
| Q1 (≤4.34) |  | Reference |  |
| Q2 (>4.34 to ≤4.71) |  | 1.07 (0.81, 1.41) | 0.630 |
| Q3 (>4.71 to ≤5.13) |  | 1.12 (0.85, 1.48) | 0.416 |
| Q4 (>5.13) |  | 1.29 (0.97, 1.71) | 0.082 |
| P for trend |  |  | 0.080 |
| Adjusted for BMI^b^ |  |  |  |
| CTI (per 1 unit) |  | 1.24 (1.03, 1.49) | 0.021 |
| CTI quartile |  |  |  |
| Q1 |  | Reference |  |
| Q2 |  | 0.97 (0.71, 1.32) | 0.837 |
| Q3 |  | 1.16 (0.86, 1.58) | 0.329 |
| Q4 |  | 1.30 (0.95, 1.78) | 0.096 |
| *P* for trend |  |  | 0.052 |

Abbreviations: CTI: C-reactive protein-triglyceride-glucose index; HR: hazard ratio; CI: confidence interval; Q1: first quartile; Q2: second quartile; Q3: third quartile; Q4: fourth quartile; BMI: body mass index.

^a^: The model was adjusted for sex, age (continuous), residence, education level, annual income, and marital status, smoking status, alcohol consumption, physical activity, hypertension, diabetes, and cardiovascular disease.

^b^: The model was adjusted for sex, age (continuous), residence, education level, annual income, and marital status, smoking status, alcohol consumption, physical activity, and BMI (linear and quadratic terms). The analysis including BMI was conducted among 8,605 participants due to missing BMI information.

**Table S3.** Association of the CTI with asthma incidence using Cox proportional hazards models adjusted for inflammatory biomarkers

| Characteristics |  | HR (95% CI) | *P* value |
| --- | --- | --- | --- |
| CTI (per 1 unit) |  | 1.21 (1.03, 1.42) | 0.023 |
| CTI quartile |  |  |  |
| Q1 (≤4.34) |  | Reference |  |
| Q2 (>4.34 to ≤4.71) |  | 1.07 (0.81, 1.42) | 0.630 |
| Q3 (>4.71 to ≤5.13) |  | 1.14 (0.86, 1.50) | 0.364 |
| Q4 (>5.13) |  | 1.30 (0.99, 1.72) | 0.059 |
| *P* for trend |  |  | 0.053 |

Abbreviations: CTI: C-reactive protein-triglyceride-glucose index; HR: hazard ratio; CI: confidence interval; Q1: first quartile; Q2: second quartile; Q3: third quartile; Q4: fourth quartile.

This model was adjusted for sex, age (continuous), residence, education level, annual income, and marital status, smoking status, alcohol consumption, physical activity, hypertension, ln-transformed white blood cell count, and ln-transformed platelet count.

**T****able S4.** Association of the CTI with asthma incidence using Cox proportional hazards models after excluding participants with chronic lung diseases at baseline

| Characteristics |  | HR (95% CI) | *P* value |
| --- | --- | --- | --- |
| CTI (per 1 unit) |  | 1.21 (1.00, 1.47) | 0.049 |
| CTI quartile |  |  |  |
| Q1 (≤4.34) |  | Reference |  |
| Q2 (>4.34 to ≤4.71) |  | 1.14 (0.82, 1.60) | 0.439 |
| Q3 (>4.71 to ≤5.13) |  | 1.18 (0.85, 1.65) | 0.328 |
| Q4 (>5.13) |  | 1.35 (0.97, 1.88) | 0.079 |
| *P* for trend |  |  | 0.083 |

Abbreviations: CTI: C-reactive protein-triglyceride-glucose index; HR: hazard ratio; CI: confidence interval; Q1: first quartile; Q2: second quartile; Q3: third quartile; Q4: fourth quartile.

This model was adjusted for sex, age (continuous), residence, education level, annual income, and marital status, smoking status, alcohol consumption, physical activity, hypertension, diabetes, and cardiovascular disease.

**Table S5.** Association of the CTI with asthma incidence using Cox proportional hazards models using an alternative formula

| Characteristics | Model 1 | |  | Model 2 | |  | Model 3 | |
| --- | --- | --- | --- | --- | --- | --- | --- | --- |
|  | HR (95% CI) | *P* value |  | HR (95% CI) | *P* value |  | HR (95% CI) | *P* value |
| CTI (per 1 unit) | 1.12 (1.00, 1.24) | 0.043 |  | 1.14 (1.02, 1.27) | 0.019 |  | 1.13 (1.01, 1.26) | 0.031 |
| CTI quartile |  |  |  |  |  |  |  |  |
| Q1 (≤8.17) | Reference |  |  | Reference |  |  | Reference |  |
| Q2 (>8.17 to ≤8.69) | 1.31 (1.00, 1.71) | 0.049 |  | 1.27 (0.97, 1.67) | 0.077 |  | 1.26 (0.96, 1.65) | 0.092 |
| Q3 (>8.69 to ≤9.23) | 1.09 (0.82, 1.45) | 0.537 |  | 1.08 (0.82, 1.43) | 0.585 |  | 1.06 (0.80, 1.41) | 0.676 |
| Q4 (>9.23) | 1.33 (1.02, 1.75) | 0.038 |  | 1.37 (1.04, 1.80) | 0.024 |  | 1.33 (1.01, 1.76) | 0.039 |
| *P* for trend |  | 0.128 |  |  | 0.081 |  |  | 0.122 |

Abbreviations: CTI: C-reactive protein-triglyceride-glucose index; OR: odds ratio; CI: confidence interval; Q1: first quartile; Q2: second quartile; Q3: third quartile; Q4: fourth quartile.

Model 1: unadjusted.

Model 2: adjusted for sex, age (continuous), residence, education level, annual income, and marital status.

Model 3: additionally adjusted for smoking status, alcohol consumption, and physical activity.

**Table S6.** Association between the CTI and asthma prevalence using logistic regression models

| Characteristics | Model 1 | |  | Model 2 | |  | Model 3 | |
| --- | --- | --- | --- | --- | --- | --- | --- | --- |
|  | OR (95% CI) | *P* value |  | OR (95% CI) | *P* value |  | OR (95% CI) | *P* value |
| CTI (per 1 unit) | 1.34 (1.16, 1.54) | <0.001 |  | 1.30 (1.12, 1.51) | <0.001 |  | 1.27 (1.09, 1.47) | 0.002 |
| CTI quartile |  |  |  |  |  |  |  |  |
| Q1 (≤4.34) | Reference |  |  | Reference |  |  | Reference |  |
| Q2 (>4.34 to ≤4.71) | 1.00 (0.76, 1.30) | 0.972 |  | 0.95 (0.72, 1.24) | 0.696 |  | 0.93 (0.71, 1.22) | 0.613 |
| Q3 (>4.71 to ≤5.13) | 1.09 (0.84, 1.42) | 0.526 |  | 1.03 (0.79, 1.35) | 0.829 |  | 1.00 (0.76, 1.31) | 0.991 |
| Q4 (>5.13) | 1.51 (1.18, 1.93) | 0.001 |  | 1.43 (1.11, 1.84) | 0.006 |  | 1.36 (1.06, 1.76) | 0.017 |
| *P* for trend |  | <0.001 |  |  | 0.002 |  |  | 0.009 |

Abbreviations: CTI: C-reactive protein-triglyceride-glucose index; OR: odds ratio; CI: confidence interval; Q1: first quartile; Q2: second quartile; Q3: third quartile; Q4: fourth quartile.

Model 1: unadjusted.

Model 2: adjusted for sex, age (continuous), residence, education level, annual income, and marital status.

Model 3: additionally adjusted for smoking status, alcohol consumption, and physical activity.

**Table S7** Baseline characteristics across CTI quartiles in external validation population

| Characteristics | | Overall  (n = 554) | CTI quartiles (%) | | | | *P* value |
| --- | --- | --- | --- | --- | --- | --- | --- |
|  |  |  | Q1 (n = 139) | Q2 (n = 139) | Q3 (n = 138) | Q4 (n = 138) |  |
| Sex | Male | 254 (45.8) | 54 (38.8) | 59 (42.4) | 61 (44.2) | 80 (58.0) | 0.008 |
|  | Female | 300 (54.2) | 85 (61.2) | 80 (57.6) | 77 (55.8) | 58 (42.0) |  |
| Age (years, continuous) |  | 67.98 (12.31) | 66.09 (12.18) | 67.76 (11.68) | 68.97 (13.06) | 69.12 (12.18) | 0.145 |
| Age (years, categorical) | 18–44 | 30 (5.4) | 8 (5.8) | 7 (5.0) | 8 (5.8) | 7 (5.1) | 0.333 |
|  | 45–59 | 68 (12.3) | 24 (17.3) | 18 (12.9) | 16 (11.6) | 10 (7.2) |  |
|  | ≥60 | 456 (82.3) | 107 (77.0) | 114 (82.0) | 114 (82.6) | 121 (87.7) |  |
| Residence | Urban | 246 (44.4) | 58 (41.7) | 58 (41.7) | 61 (44.2) | 69 (50.0) | 0.465 |
|  | Rural | 308 (55.6) | 81 (58.3) | 81 (58.3) | 77 (55.8) | 69 (50.0) |  |
| Education level | No formal education or primary school | 21 (3.8) | 5 (3.6) | 5 (3.6) | 6 (4.3) | 5 (3.6) | 0.888 |
|  | Middle school | 385 (69.5) | 93 (66.9) | 94 (67.6) | 96 (69.6) | 102 (73.9) |  |
|  | High school | 120 (21.7) | 33 (23.7) | 35 (25.2) | 27 (19.6) | 25 (18.1) |  |
|  | College or above | 28 (5.1) | 8 (5.8) | 5 (3.6) | 9 (6.5) | 6 (4.3) |  |
| Marital status | Married | 547 (98.7) | 136 (97.8) | 137 (98.6) | 137 (99.3) | 137 (99.3) | 0.667 |
|  | Unmarried or other | 7 (1.3) | 3 (2.2) | 2 (1.4) | 1 (0.7) | 1 (0.7) |  |
| Current drinking | No | 525 (94.8) | 133 (95.7) | 132 (95.0) | 131 (94.9) | 129 (93.5) | 0.870 |
|  | Yes | 29 (5.2) | 6 (4.3) | 7 (5.0) | 7 (5.1) | 9 (6.5) |  |
| Current smoking | No | 438 (79.1) | 119 (85.6) | 116 (83.5) | 107 (77.5) | 96 (69.6) | 0.005 |
|  | Yes | 116 (20.9) | 20 (14.4) | 23 (16.5) | 31 (22.5) | 42 (30.4) |  |
| Hypertension | No | 457 (82.5) | 116 (83.5) | 113 (81.3) | 120 (87.0) | 108 (78.3) | 0.279 |
|  | Yes | 97 (17.5) | 23 (16.5) | 26 (18.7) | 18 (13.0) | 30 (21.7) |  |
| Diabetes | No | 493 (89.0) | 128 (92.1) | 117 (84.2) | 132 (95.7) | 116 (84.1) | 0.002 |
|  | Yes | 61 (11.0) | 11 (7.9) | 22 (15.8) | 6 (4.3) | 22 (15.9) |  |
| Cardiovascular disease | No | 508 (91.7) | 122 (87.8) | 125 (89.9) | 133 (96.4) | 128 (92.8) | 0.056 |
|  | Yes | 46 (8.3) | 17 (12.2) | 14 (10.1) | 5 (3.6) | 10 (7.2) |  |

Abbreviations: CTI: C-reactive protein-triglyceride-glucose index; Q1: first quartile; Q2: second quartile; Q3: third quartile; Q4: fourth quartile.

**Table S8** Baseline characteristics across asthma in external validation population

| Characteristics |  | Overall | No asthma | Asthma | *P* value |
| --- | --- | --- | --- | --- | --- |
|  |  | (n = 554) | (n = 277) | (n = 277) |  |
| Sex | Male | 254 (45.8) | 127 (45.8) | 127 (45.8) | 1.000 |
|  | Female | 300 (54.2) | 150 (54.2) | 150 (54.2) |  |
| Age (years, continuous) |  | 67.98 (12.31) | 68.04 (12.02) | 67.93 (12.62) | 0.920 |
| Age (years, categorical) | 18–44 | 30 (5.4) | 14 (5.1) | 16 (5.8) | 0.828 |
|  | 45–59 | 68 (12.3) | 36 (13.0) | 32 (11.6) |  |
|  | ≥60 | 456 (82.3) | 227 (81.9) | 229 (82.7) |  |
| Residence | Urban | 246 (44.4) | 140 (50.5) | 106 (38.3) | 0.005 |
|  | Rural | 308 (55.6) | 137 (49.5) | 171 (61.7) |  |
| Education level | No formal education or primary school | 21 (3.8) | 12 (4.3) | 9 (3.2) | 0.633 |
|  | Middle school | 385 (69.5) | 187 (67.5) | 198 (71.5) |  |
|  | High school | 120 (21.7) | 65 (23.5) | 55 (19.9) |  |
|  | College or above | 28 (5.1) | 13 (4.7) | 15 (5.4) |  |
| Marital status | Married | 547 (98.7) | 272 (98.2) | 275 (99.3) | 0.447 |
|  | Unmarried or other | 7 (1.3) | 5 (1.8) | 2 (0.7) |  |
| Current drinking | No | 525 (94.8) | 261 (94.2) | 264 (95.3) | 0.703 |
|  | Yes | 29 (5.2) | 16 (5.8) | 13 (4.7) |  |
| Current smoking | No | 438 (79.1) | 233 (84.1) | 205 (74.0) | 0.005 |
|  | Yes | 116 (20.9) | 44 (15.9) | 72 (26.0) |  |
| Hypertension | No | 457 (82.5) | 219 (79.1) | 238 (85.9) | 0.044 |
|  | Yes | 97 (17.5) | 58 (20.9) | 39 (14.1) |  |
| Diabetes | No | 493 (89.0) | 241 (87.0) | 252 (91.0) | 0.175 |
|  | Yes | 61 (11.0) | 36 (13.0) | 25 (9.0) |  |
| Cardiovascular disease | No | 508 (91.7) | 253 (91.3) | 255 (92.1) | 0.878 |
|  | Yes | 46 (8.3) | 24 (8.7) | 22 (7.9) |  |
